# Depicting developing trend and core knowledge of hip fracture research: a bibliometric and visualised analysis

**DOI:** 10.1186/s13018-021-02292-x

**Published:** 2021-03-04

**Authors:** Guanrong Peng, Zhenhua Guan, Yunfei Hou, Jiaxiang Gao, Wenqun Rao, Xianyun Yuan, Jiusheng Guo, Xiaohua Huang, Zhangrong Zhong, Jianhao Lin

**Affiliations:** 1 The First Clinical Medical School, Guangzhou University of Chinese Medicine, Jichang Road 12#, District Baiyun, Guangzhou, Guangdong Province China; 2 Yudu People’s Hospital, No. 2, Huancheng North Road, Gongjiang Town, Yudu, 342300 Jiangxi Province China; 3 Peking University People’s Hospital, No. 11, Xizhimen South Street, Beijing, 100044 Xicheng District China

## Abstract

**Background:**

Hip fracture is common and carries high morbidity and mortality; thus, it has become a vital concern. We aim to analyse the present status, worldwide trends in hip fracture and state of clinical research.

**Methods:**

Publications from 2000 to 2019 were retrieved from the Web of Science database and analysed using a bibliometric methodology. VOSviewer software was utilised for analysis.

**Results:**

In total, 6139 publications were included, and publications increased annually from 152 in 2000 to 592 in 2019. U.S. researchers have produced the most publications, the highest H-index and the greatest number of citations. *Osteoporosis International* has published the most papers on the topic. Leading researchers, contributing institutions, their cooperative relationships and scientific masterpieces have been identified. The publications can be divided into five clusters: ‘mortality’, ‘surgical management’, ‘rehabilitation’, ‘osteoporosis’ and ‘epidemiology’. A clear developing trend was described, which began with fracture epidemiology and prevention, transitioned to perioperative management, orthogeriatric care and patient safety and then to functional recovery, disease burden and national audits in recent times.

**Conclusions:**

Hip fractures result in conditions that extend far beyond orthopaedics concerning epidemiology and preventive medicine, internal medicine and endocrinology, as well as critical care and gerontology. Interest, research and publications are on the rise.

## Background

With an ageing population around the world, hip fracture has become a vital concern. The number of hip fractures is anticipated to increase from 1.26 million in 1990 to 4.5 million by 2050. Although the age-standardised rate is slowly decreasing in many nations, the growing number of elderly is outpacing it [1].

Amongst all osteoporotic fractures, hip fracture carries the highest morbidity and mortality [2]. All-cause mortality and excess mortality after hip fracture are greater than that of age-matched controls even after two decades of follow-up [3]. Fracture survivors encounter substantially worse mobility, independence, overall health and quality of life [4]. Even so, worldwide trends in hip fractures have not been well analysed.

Bibliometric analysis is a feasible strategy to summarise and anticipate the research trends qualitatively and quantitatively by evaluating the studies of major authors, journals, institutes and nations [5]. Additionally, it makes contributions to clinical policy-making and guideline development [6]. The objective of this study is to analyse the present status of hip fracture and trends in clinical research.

## Methods

### Data source

Whilst many databases could satisfy the need for analysis at a global level [7], we selected the Web of Science (WoS) and Science Citation Index-Expanded for this evaluation. These databases cover more than 12,000 international scientific journals of greatest impact and quality, offering detailed information on publications [8].

### Search strategy

The search strategy was as follows: (title=hip AND title=fracture*). We excluded pathological fractures caused by bone tumours and fractures following any type of hip arthroplasty. We included publications from 2000 to 2019. On June 21, 2020, we identified and retrieved 355 reviews and 5784 articles.

### Data extraction

Information on all identified publications—including title, author, publication year, contributing nations, affiliations, journal, keywords and abstract—was downloaded. Two authors independently browsed and extracted data from the eligible publications.

### Bibliometric analysis

The basic characteristics of publications are an intrinsic function of WoS. The H-index is described as the value according to a scholar or scientist who has published H papers, each of which has been cited by other studies no less than H times [9]. Therefore, the H-index identifies the number of publications by each researcher and all relevant citations, enabling evaluation of an author’s productivity and the impact of the published research [10].

### Visualised analysis

VOSviewer (Leiden University, Leiden, The Netherlands) is a programme for creating and visualising bibliometric networks [11]. In this particular study, VOSviewer was used for coauthorship, co-citation and co-occurrence analysis. In the network map developed by VOSviewer, various nodes represented different elements, including authors, countries, institutions and keywords. The size of the nodes reflected the number of publications or frequency [12]. The links between nodes represented the associations, including co-authorship or co-occurrence, whilst the colour of the node/lines reflected diverse clusters or years [13]. The strength of the link was presented as the total link strength (TLS).

Co-authorship analysis illustrates the connection amongst items in line with the number of co-authored papers, which is an effective tool to evaluate collaboration trends and to identify leading researchers, nations, and organisations [14]. Co-occurrence analysis illustrates the connection of keywords according to the quantity of publications where they were found together [15]. This analysis explores popular subjects and research directions; thus, it is a crucial indicator of developments in a specific research area. A repeated co-occurrence analysis was conducted using a second dataset with a narrower time period, 2018-2020, whilst other conditions (selected database, search strategy, exclusion criteria and document types) remained the same. Keywords with a high frequency of use in 2018-2020 were compared with those generated from the analysis of 2000-2019. These analyses captured the trend in hip fracture research.

## Results

### Quantity of global publications

**Fig. 1:**
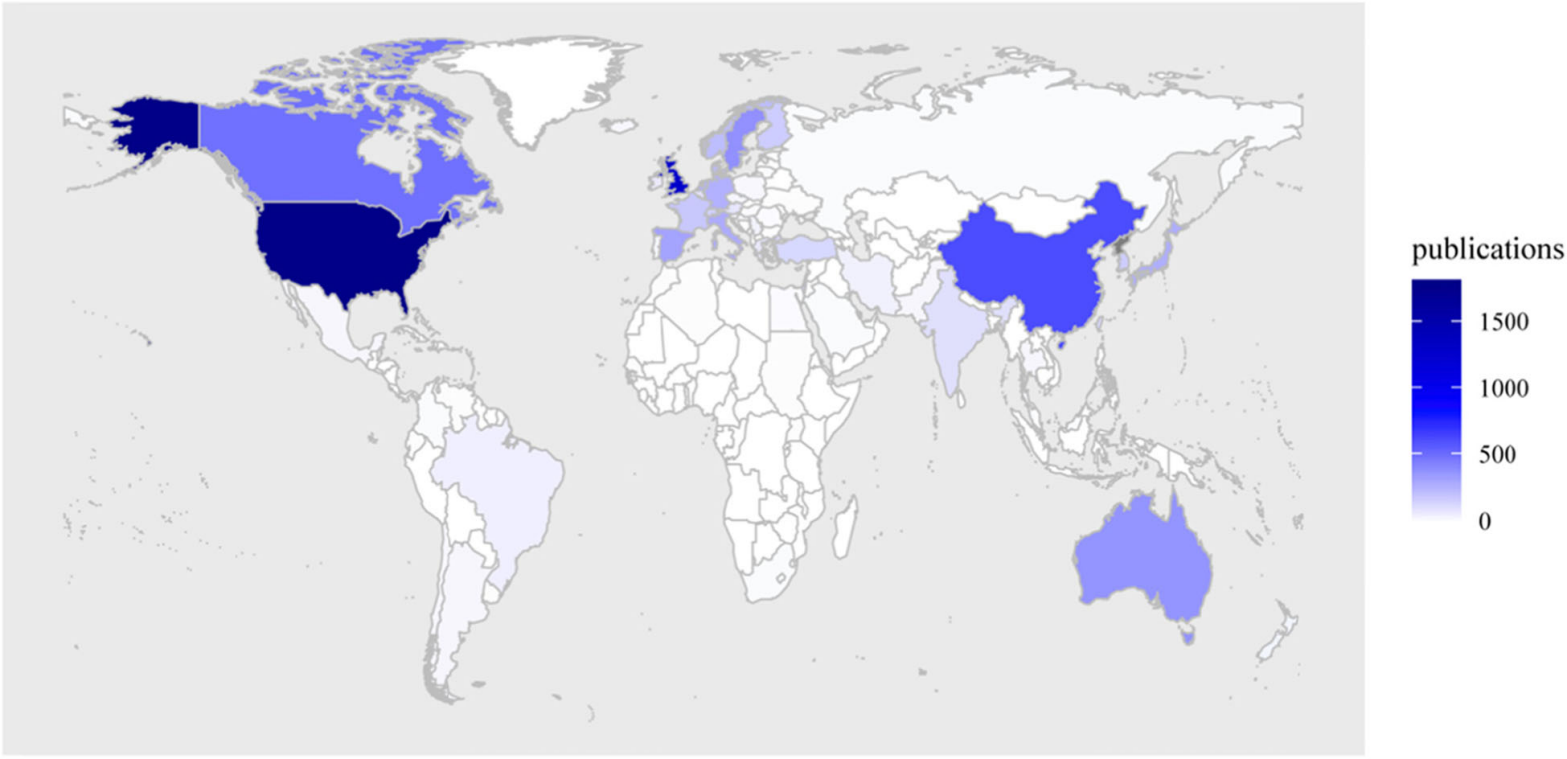
The distribution world map of hip fracture research

In total, 6139 publications (355 reviews and 5784 articles) were included in this study. Over the past two decades, the number of topical publications increased yearly, from 152 in 2000 to 592 in 2019, as shown in Table 1. Most of the manuscripts were published in 2019 (592, 9.6%), and a total of 86 nations and regions published relevant articles/reviews. The countries that made the greatest contributions are presented in Table 1. A distribution world map of hip fracture research is shown in Fig. 1.

**Table 1:** The quantity of hip fracture research in terms of year and country

Year (publications)		Country (publications)	
2019 (592)	2009 (272)	USA (1766)	Netherlands (263)
2018 (496)	2008 (228)	UK (1269)	Denmark (230)
2017 (506)	2007 (214)	China (615)	Norway (221)
2016 (466)	2006 (217)	Canada (487)	France (182)
2015 (407)	2005 (215)	Sweden (379)	Israel (174)
2014 (393)	2004 (170)	Australia (365)	Finland (167)
2013 (378)	2003 (163)	Spain (311)	South Korea (166)
2012 (331)	2002 (162)	Italy (282)	Switzerland (149)
2011 (349)	2001 (113)	Japan (271)	Turkey (124)
2010 (315)	2000 (152)	Germany (267)	India (101)

### Quality of publications from each country/region

The total number of citations and the H-index reflect the quality of publications and academic impact of one country [16]. The USA had the highest number of total citations (64,086), whilst the UK ranked second (28,505), followed by Canada (17,001), Sweden (13,387) and Australia (11,386). The same trend was present for the H index: USA (118), UK (76), Canada (61), Sweden (57) and Australia (53). Publications from Switzerland had the highest average citation frequency (45.46), followed by France (41.33), Netherlands (40.63), the USA (38.68), Canada (36.64) and Denmark (36.58).

### Analysis of global publications

#### Journals

*Osteoporosis International* published 459 articles/reviews, outranking other journals with the most publications. *Injury-International Journal of the Care of the Injured* was second with 347 publications. There were 165 papers published in the *Journal of Bone and Mineral Research*, 157 in the *Journal of Orthopaedic Trauma* and 134 in the *Journal of the American Geriatrics Society*. The top 10 journals with the most publications are listed in Table 2.

**Table 2:** Leading journals, authors and institutions of publications related to hip fracture research

Journal (publications)	Author (publications)	Institution (publications)
OSTEOPOROSIS INT (459)	Magaziner J (93)	Univ Maryland (145)
Injury (347)	Parker MJ (79)	Univ Pittsburgh (112)
J BONE MINER RES (165)	Cauley JA (58)	Univ California San Francisco (109)
J ORTHOP TRAUMA (157)	Di Monaco M (48)	Karolinska Inst (101)
J AM GERIATR SOC (134)	Cooper C (44)	Univ Oxford (99)
J ARTHROPLASTY (128)	Kanis JA (42)	Harvard Univ(98)
BONE (116)	Cummings SR (41)	Univ Toronto (97)
J BONE JOINT SURG AM (113)	Bhandari M (40)	Tel Aviv Univ (91)
INT ORTHOP (109)	Ha YC (40)	Univ Oslo (88)
ARCH ORTHOP TRAUM SU (93)	Ensrud KE (39)	McMaster Univ (86)

#### Research orientation

**Fig. 2:**
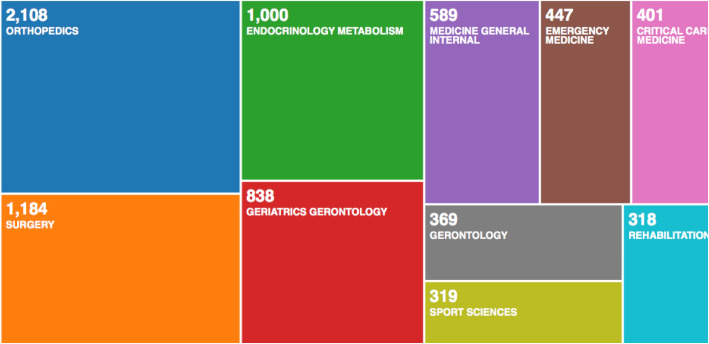
The top 10 research orientations and the number of publications in each orientation

The top 10 research orientations related to hip fracture are shown in Fig. 2. By far, the most predominant areas of research were orthopaedics (2108 papers), surgery (1184 papers), sport science (1586 papers), engineering (432 papers) and general internal medicine (589 papers).

#### Authors

The top 10 authors with the greatest number of publications are listed in Table 2. In total, these authors have published 1151 articles/reviews in the past 20 years. Magaziner J from the USA outranked other researchers with 93 publications, followed by Parker MJ from the UK with 79 papers and Cauley JA from the USA with 58 papers. It is noteworthy that we included all authors in the analysis, regardless of their relative contribution (first author, correspondence author or co-author).

#### Institution output

As presented in Table 2, the University of Maryland had the greatest number of publications, with 145 papers, followed by the University of Pittsburgh (112 papers), and then the University of California, San Francisco (109 papers).

#### Top 10 most-cited articles and top 10 articles with greatest number of citations in a given year

The mean number of citations per publication was 25.9. Table 3 demonstrates the top 10 most-cited articles regarding hip fracture. The most highly cited article was ‘Effect of risedronate on the risk of hip fracture in elderly women’, published in the *New England Journal of Medicine (NEJM)* by McClung et al. [17], with 1321 citations on WOS. Table 4 lists the top 10 articles on hip fracture with the greatest number of citations in a given year, amongst which the article ‘Zoledronic acid and clinical fractures and mortality after hip fracture’, published in the *NEJM* by Lyles et al. [18], ranked first with 75.5 citations.

**Table 3:** Top 10 most-cited publications in hip fracture research

Rank	Title of the publication/first author/publishing year/publishing journal	Citation rate
1	Effect of risedronate on the risk of hip fracture in elderly women/McClung/2001/The New England Journal of Medicine	1321
2	Zoledronic acid and clinical fractures and mortality after hip fracture/Lyles/2007/The New England Journal of Medicine	1057
3	Predictive value of BMD for hip and other fractures/Olof/2005/Journal of Bone and Mineral Research	866
4	Long-term proton pump inhibitor therapy and risk of hip fracture/Yang/2006/The Journal of the American Medical Association	745
5	Incidence and Mortality of Hip fractures in the United States/Braur/2009/The Journal of the American Medical Association	695
6	The use of clinical risk factors enhances the performance of BMD in the prediction of hip and osteoporotic fractures in men and women/Kanis/2007/Osteoporosis International	690
7	Reducing delirium after hip fracture: A randomized trial/Marcantonio/2001/Journal of American Geriatric Society	689
8	Effect of comorbidities and postoperative complications on mortality after hip fracture in elderly people: prospective observational cohort study/Roche/2005/British Medical Journal	658
9	Meta-analysis: Excess Mortality After Hip fracture Among Older Women and Men/Patrick/2010/Annals of Internal Medicine	600
10	A systematic review of hip fracture incidence and probability of fracture worldwide/Kanis/2012/Osteoporosis International	530

**Table 4:** Top 10 publications with the largest annual citations

Rank	Title of the publication/first author/publishing year/publishing journal	Annual citations
1	Zoledronic acid and clinical fractures and mortality after hip fracture/Lyles/2007/The New England Journal of Medicine	75.5
2	Effect of risedronate on the risk of hip fracture in elderly women/McClung/2001/The New England Journal of Medicine	66.05
3	A systematic review of hip fracture incidence and probability of fracture worldwide/Kanis/2012/Osteoporosis International	58.89
4	Incidence and Mortality of Hip fractures in the United States/Braur/2009/The Journal of the American Medical Association	57.92
5	Meta-analysis: Excess Mortality After Hip fracture Among Older Women and Men/Patrick/2010/Annals of Internal Medicine	54.55
6	Predictive value of BMD for hip and other fractures/Olof/2005/Journal of Bone and Mineral Research	54.13
7	Long-term proton pump inhibitor therapy and risk of hip fracture/Yang/2006/The Journal of the American Medical Association	49.67
8	The use of clinical risk factors enhances the performance of BMD in the prediction of hip and osteoporotic fractures in men and women/Kanis/2007/Osteoporosis International	49.29
9	Effect of comorbidities and postoperative complications on mortality after hip fracture in elderly people: prospective observational cohort study/Roche/2005/British Medical Journal	41.13
10	Secular trends in the incidence of hip and other osteoporotic fractures/Cooper/2011/Osteoporosis International	41.1

### Visualised analysis

#### Coauthorship analysis

##### Authors

**Fig. 3:**
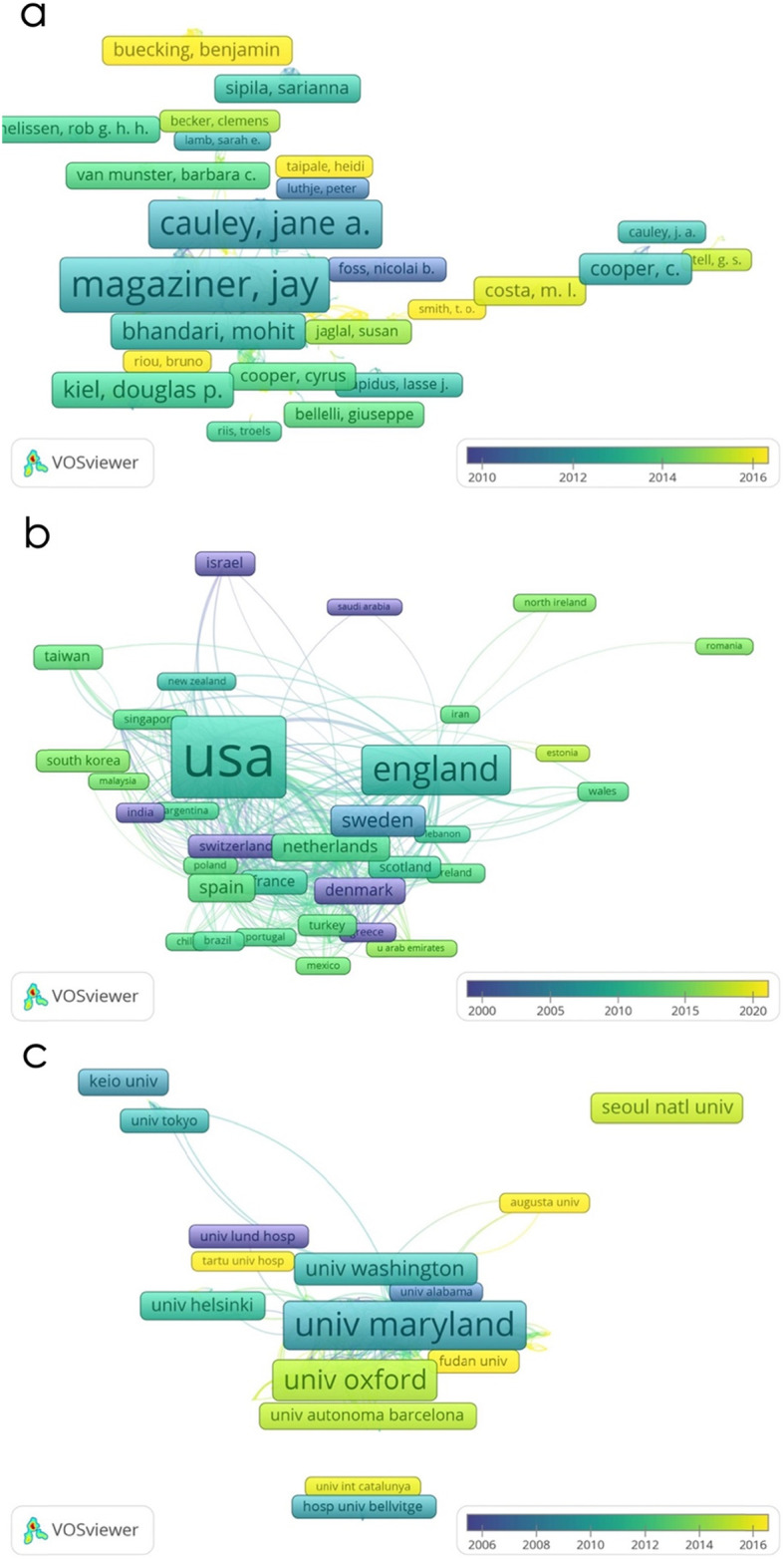
Coauthorship analysis in hip fracture research. **a** Mapping of the co-authorship analysis amongst 480 identified authors. **b** Mapping of 54 identified countries. **c** Mapping of 622 institutions

As presented in Fig. 3a, a total of 480 authors with a minimum of 5 publications were identified and analysed. The top five authors with the greatest TLS were Magaziner J (TLS = 372 times), Cauley JA (TLS = 189 times), Ensrud K (TLS = 127 times), Cumming S (TLS = 124 times) and Orwig D (TLS = 118 times).

##### Countries and regions

A total of 54 countries and regions with a minimum of 5 publications were identified. The top five countries and regions with the largest TLSs were as follows: the USA (TLS = 683 times), the UK (TLS = 595 times), Canada (TLS = 341 times), Sweden (TLS = 287 times) and the Netherlands (TLS = 280 times), as shown in Fig. 3b.

##### Institutions

As presented in Fig. 3c, 622 institutions were included with a minimum of 5 publications. The University of Pittsburgh (TLS = 395 times), University of Maryland (TLS = 332 times), University of California San Francisco (TLS =318 times), Harvard University (TLS = 260 times) and McMaster University (TLS = 249 times) were the top five institutions with the greatest TLS.

##### Co-occurrence analysis

**Fig. 4:**
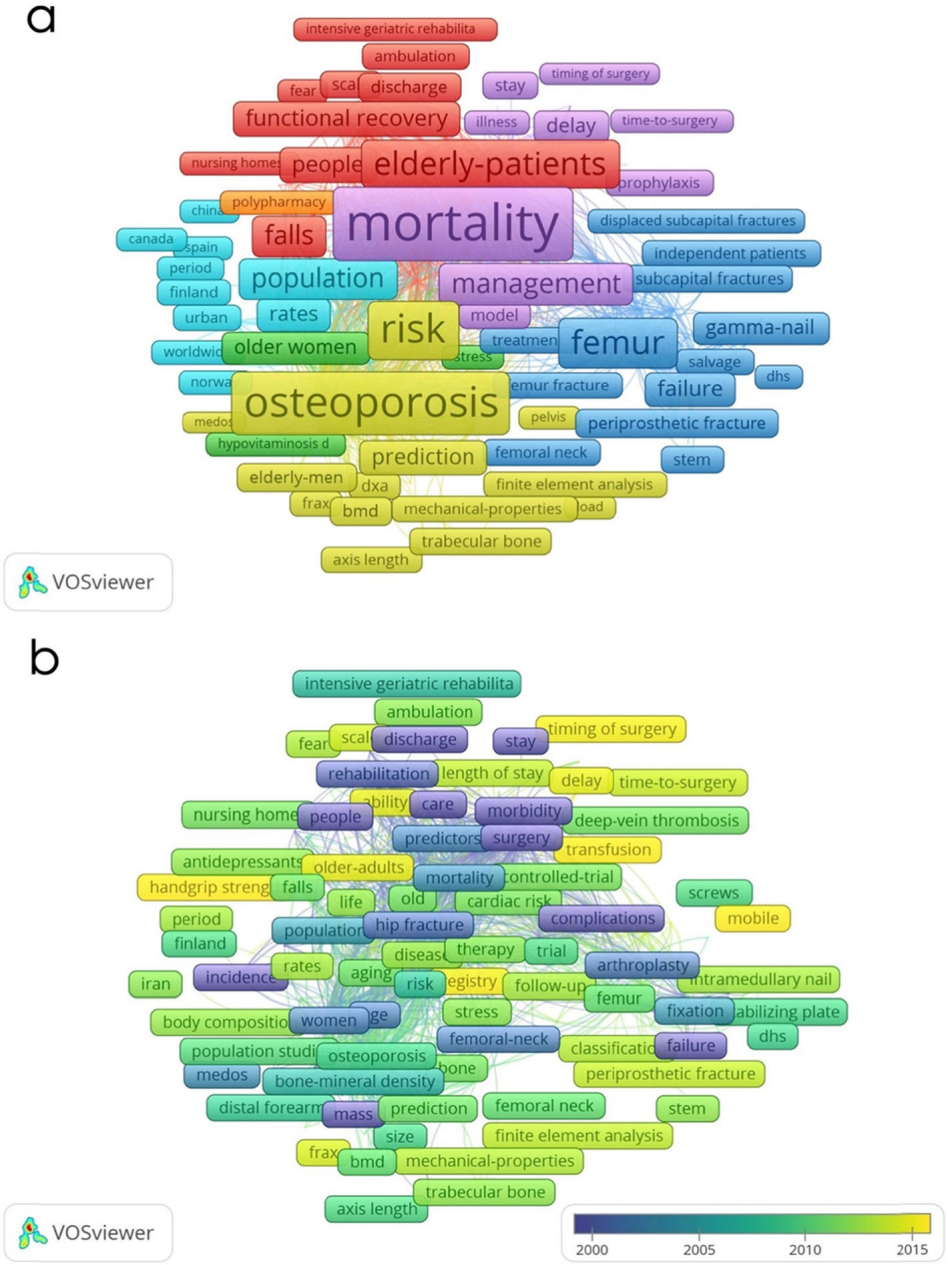
Co-occurrence analysis on hip fracture research. **a** Mapping of keywords in hip fracture research; the size of nodes represents the frequency, whilst the lines between nodes reflect the co-occurrence relationship. A total of 1458 included keywords were divided into five clusters: ‘mortality study’ (colour purple), ‘surgical management study’ (colour deep blue), ‘rehabilitation study’ (colour red), and ‘epidemiology study’ (colour light blue). **b** Distribution of keywords according to the time of appearance. The colour blue indicates the keywords that appeared earlier, whereas the colour red reflects the later occurrence

Keywords utilised more than five times in the publications were recognised and analysed via VOSviewer. As presented in Fig. 4a by different colours, the 1458 keywords could be divided into approximately 5 study clusters: ‘mortality’, ‘surgical management’, ‘rehabilitation’, ‘osteoporosis’ and ‘epidemiology’. Within the ‘mortality study’ cluster, frequent keywords were morbidity, survival, operative delay, complications and blood loss. Within the ‘surgical management study’, frequent keywords were arthroplasty, fixation, follow-up, outcomes and failure. In ‘rehabilitation study’, keywords were nursing home, geriatric rehabilitation, cognitive impairment and delirium. Within ‘osteoporosis study’, keywords were bone mineral density (BMD), ageing, risk prediction and trabecular bone. Within ‘epidemiology study’, frequent keywords were incidence, rates, population and risk factors. The overlay visualisation map of the co-occurrence analysis, with items denoted by colours in accordance with the average time period when the keywords occurred [19], is illustrated in Fig. 4b. Blue indicates keywords that appeared earlier, whilst red indicates keywords that appeared later. Before 2010, keywords included ‘rehabilitation’, ‘surgery’, ‘morbidity’, ‘mortality’ and ‘complications’, coded in blue, occurred earlier, as they were key and elemental aspects in hip fracture management and research, whilst after 2010, as the research was conducted in a more detailed manner, keywords such as ‘timing/delay of surgery’, ‘transfusion’, ‘registry’ and ‘mobile’, started to occur.

A repeated co-occurrence analysis using a second dataset based on the time period 2018-2020 demonstrated keywords with high occurrence in more recent years then compared them with those generated using the primary dataset. These comparisons are shown in Table 5. From 2018 to present the keywords ‘surgery’ ‘outcomes’ ‘elderly patients’ ‘management’ and ‘complications’ occurred more often than they did in the past.

**Table 5:** Top 10 key words with the highest occurrences in different time period: 2018-2020 vs 2000-2019

Rank	2018-2020	2000-2019
1	mortality	mortality
2	surgery^a^	osteoporosis
3	risk	risk
4	outcomes^a^	women
5	osteoporosis	surgery
6	elderly-patients^a^	outcomes
7	management^a^	Bone-mineral density
8	women	Elderly-patients
9	complications^a^	epidemiology
10	risk-factors	Risk-factors

^a^Higher rankings compared to which at the time period 2000-2019

## Discussion

In this study, the current status and global trends of hip fracture research were delineated. The annual quantity of publications has gradually increased. Leading researchers, institutions, countries and their cooperative relationships have been identified, and important publications with high numbers of citations have been highlighted.

Utilising co-occurrence cluster analysis, we presented a network map of co-occurrence relationships by analysing keywords found in relevant studies. In total, five potential research orientations were identified. These results suggest that hip fractures, especially in older individuals, lead to conditions that extend far beyond the orthopaedic injury, with consequences in the aspects of epidemiology and preventive medicine, internal medicine and endocrinology, as well as critical care and gerontology. This is confirmed in Fig. 2. Different colours in the overlay visualisation map of the co-occurrence analysis represent the relevant year of publication. Nodes of various colours (from blue to red) could all be found with substantial densities in the five clusters, which suggested that a pattern of balanced development existed in these five directions. Additionally, each direction was also undergoing changes in research hot topics, suggesting that research was diversifying.

Most of the highly cited studies were published before 2010, as prior studies inherently have more time to accumulate citations than more recently published manuscripts. These earlier studies mainly focused on osteoporosis management, fracture epidemiology and prevention, as well as perioperative management and patient safety.

### Fracture prevention and medication therapy

The most-cited report was published by McClung in 2001 describing the protective effects of risedronate, which substantially minimised hip fracture risk amongst older females with established osteoporosis [17]. The results of this large trial (9331 female patients) also demonstrated the value of BMD measurements in identifying women for whom medication therapy is adequate. Similarly, a double-blinded randomised controlled trial (RCT) discovered that an annual infusion of zoledronic acid within 90 days following surgical fixation was associated with a reduction in a new clinical fracture rate together with improved survival [18]. Conversely, a nested case-control study carried out utilising the General Practice Research Database (1987-2003) in the UK discovered that long-term proton pump inhibitor therapy, especially at high doses, was associated with an elevated hip fracture risk [20].

### Epidemiology, mortality and long-term survival

Other research centred on epidemiology. In the USA, hip fracture rates and consequent mortality amongst individuals 65 years and older decreased with the usage of bisphosphonates, whilst comorbidities amongst patients with hip fractures increased [21]. An additional systematic review (SR) reported that the age-standardised hip fracture rates were accessible for 63 countries [22]. There was a greater than tenfold variation in the risks between nations. High-risk regions for men were Taiwan, Austria, the USA (Whites), Switzerland, Norway, Sweden and Denmark. Low-risk regions included Tunisia, Oceania, the Latin American countries of Ecuador and Colombia and several European countries (Spain, Poland, Romania, France and Turkey), China, Lebanon, the Philippines and the USA (Blacks). The basic pattern of fracture likelihood in women was comparable to that in men [22]. Another study noted that the risk could be predicted by BMD and clinical risk factors (CRFs). The prediction model, with the combined use of CRFs and BMD rather than BMD alone, could be improved with a greater gradient of risk (risk ratio/standard deviation change in risk score) from 3.7/SD to 4.2/SD [23].

Regarding mortality, a meta-analysis reported that older adults have a five- to eightfold higher risk for all-cause mortality throughout the first 3 months after hip fracture. With the use of life-table methods, the investigators estimated that an American white woman who has a hip fracture at age 80 has an excess annual mortality of 8%, 11%, 18% and 22% at 1, 2, 5 and 10 years after injury, respectively. The corresponding figures for an American white man were 18%, 22%, 26% and 20% [24]. Von Friesendorff and colleagues followed 1013 hip fracture patients and 2026 matched community controls for 22 years, which is the longest follow-up duration amongst similar studies. From a remaining lifetime perspective, all-cause and excess mortality after hip fracture was higher even over two decades of follow-up. Cardiovascular diseases and pneumonia reduced life expectancy for the remaining lifetime [3].

### Perioperative management and orthogeriatric collaborative care

A prospective cohort study reported that in elderly patients, the existence of three or more comorbidities would be the strongest CRF for mortality within the first month postoperatively. Pneumonia and heart failure were again the most common early postoperative complications and resulted in increased mortality. These groups offer an apparent target for specialised medical evaluation [25]. On surgical timing, earlier surgery (less than 72 h) was associated with a reduced risk of death and lower rates of complications [26]. Another RCT noted that proactive geriatric consultation was effectively applied with good adherence after surgery. It diminished delirium by more than one-third and reduced severe delirium by more than half. The trial provided strong preliminary evidence that proactive geriatric consultation played a crucial role in acute hospital management [27]. A later study reported that immediate admission of patients age 70 years or older to comprehensive geriatric care in a dedicated ward improved mobility at 4 months compared with usual orthopaedic care alone [4].

Since 2010, research has concentrated more on patient rehabilitation, national audit and registry studies.

### Patient rehabilitation, functional outcome and medical and economic burden

Fracture has a considerable impact on older individuals’ medium- to longer-term capabilities, physical function, quality of life and need for accommodations. Only 40% to 60% of patients recovered their pre-fracture level of mobility, whilst 40%-70% regained their level of independence for fundamental activities of daily living (ADL) [4]. Reported in a cohort study with data from an RCT, approximately 30% (556/1857) of the previously ambulatory cohort were not ambulating 10 feet without human assistance 60 days after randomisation [28]. Only 24% of patients returned to their baseline ADL at 3 months after hip fracture treatment, and only 29% did so 12 months postoperatively [29]. Several factors that could impede patients returning to prefracture status have been identified, including late operation after 36 h [30], low-volume skilled nursing facilities (24 admissions/year) [31], older age, preexisting dementia, admission from a nursing home, cardiovascular disease, higher American Society of Anaesthesiologists (ASA) risk score [28] and longer length of hospital stay [29]. These results suggest that great medical and potential economic burdens exist for hip fracture survivors. Medical expenses following hip fracture were high. There is a solid economic incentive to prioritise research funds towards determining the best strategies to prevent both index and subsequent hip fractures [32].

### National audit and initiative

The great burden caused by hip fracture warrants action on a greater scale, i.e. country level. The UK National Hip Fracture Database was launched in 2007 as a national collaborative, clinician-led audit initiative to enhance hip fracture care quality, which was associated with significant improvements in the care and survival of aged individuals with hip fracture. From 2007 to 2011, the early surgery rate increased from 54.5 to 71.3% and remained stable from 2003-2007. Thirty-day mortality fell from 10.9 to 8.5%. The yearly relative decrease in adjusted 30-day mortality was 1.8% per year in the time period 2003-2007, compared with 7.6% per year over 2007-2011 [33].

### Changing trends of hip fracture and its research

Analysis of secular trends in age-adjusted hip fracture rates worldwide showed differences between countries and continents. In the USA, Canada, Northern and Western Europe, Oceania, Hong Kong and Taiwan, the age-standardised fracture incidence or crude incidence is decreasing [34]. In a study conducted in France from 2002 to 2013, the incidence of hip fracture rose by 4.8% in women (from 49,287 to 51,661) and 21.8% in men (from 12,716 to 15,482) aged over 59 years. Meanwhile, the French population over 59 years increased, with a rise of 21.3% in women and 28.7% in men, resulting in a decrease in the crude incidence rates of 13.6% in women and 5.4% in men [35]. In a similar study in the USA using 2002 to 2015 Medicare data, authors reported that for women ≥ 65 years old, age-standardised hip fracture rates declined each year from 2002 (844/100,000) to 2012 (741/100,000) and then plateaued in 2013 (741/100,000) [36]. In contrast, rising rates have been reported in Southern Europe, South America and many parts of Asia [34].

Regarding patient characteristics, investigators found that hip fracture patients are becoming older and increasingly frail [37]. According to a Danish study, patients have more co-morbidities; the largest increase was seen for congestive heart failure, liver and renal disease [38]. Despite increasing frailty, the 30-day and 12-month rates of mortality fell significantly (*p* = 0.002 and 0.001, respectively) [37]. In a recent SR involving studies published in 2013-2017, hip fracture-related studies from 36 different countries were reviewed with regard to 1-year mortality rates. A total of 229,851 patients were included, with a range of 100-43,830 patients in the smallest to largest cohorts, respectively. The mean overall 1-year mortality rate declined from approximately 30% to 22.0% with a range from 2.4-34.8% [39]. The risk of reoperation has also decreased over a 10-year period [40]. These results may suggest a consistent global improvement in hip fracture care quality. Overall, hip fractures are becoming more common and more complex in an ageing and increasingly frail population, and these trends are expected to continue [37].

This research has identified a clear trend in hip fracture research over the past two decades, which began with fracture epidemiology and prevention, transitioned to perioperative management, orthogeriatric care and patient safety, and then to patient rehabilitation, disease burden and national audit studies in recent years. As the number of topical publications increases and a significant burden of hip fracture prevails, more vigorous studies can be expected. Through the results of the overlay visualisation map in co-occurrence analysis, ‘timing of surgery, registry, and patient mobility’ indicated that more and more studies after 2010 are focusing on patient safety, functional recovery and big data research. Additionally, we used a second data set from 2018 to 2020 in the co-occurrence analysis to identify keywords (i.e. ‘surgery’, ‘outcomes’, ‘elderly patients’, ‘management’ and ‘complications’) with increasing frequency in recent years, as shown in Table 5. Given the similar and interrelated results, we anticipated several hot topics in hip fracture research. (1) Optimisation of peri-operative management and complication prevention; (2) post-injury rehabilitation and care; (3 meta-analysis, registry and big data research.

This study inevitably has some limitations. First, there are intrinsic differences between the results of bibliometric analysis and real-world studies. For instance, some comparatively new publications of high quality may not attach sufficient attention due to lower citation frequency, whilst older articles have a tendency to accumulate more citations. A second limitation is the ‘obliteration by incorporation’ effect describing the bias created with citation analysis, which occurs when particular ideas become so accepted that the most original work is no longer cited [41]. Additionally, self-citing (or neglecting to cite a rival’s work) might bring in the inherent bias of ‘incomplete citing’ and ‘omission bias’.

In this study, with the usage of bibliometric and visualised analysis, hot topics in research and collaborative relationships amongst countries, authors and institutions were identified, and scientific masterpieces were reviewed. This information could provide investigators with a vivid general view within the academic field of hip fracture research. A time trend was depicted from its epidemiology, osteoporosis management and fracture prevention in the first decade of twenty-first century, to patient mortality and surgery timing in the later time period, to rehabilitation as well as national registry and audit research in the last period. This information could also guide stakeholders in prioritising funding and optimising the care of hip fracture.

## Data Availability

Data will be available upon request by the first author GP.

## Abbreviations

WoS: Web of Science
TLS: Total link strength
NEJM: New England Journal of Medicine
BMD: Bone mineral density
FU: Follow up
RCT: Randomised controlled trial
CRFs: Clinical risk factors
SR: Systematic review
ADL: Activities of daily living
ASA: American Society of Anaesthesiologists
